# Continuous Local Infection

**Published:** 1904-01-09

**Authors:** 


### CONTINUOUS LOCAL INFECTION.

Professor Rickman Godlee,[1] speaking on the above subject before the Bradford Medico-Chirurgical Society, called attention to the far-reaching effects of small collections of septic material in the body, and the grave symptoms produced by oft-repeated small doses of organic poison. He described cases of appendicitis in which when, after an attack, all the local signs had cleared up, the patients were still out of health, constipated and dyspeptic, and the removal of an apparently almost normal appendix had resulted in their regaining a condition of health and vigour to which for years they had been strangers. He described the case of a patient who had suffered from anorexia, emaciation, offensive diarrhœa, and fever, as the result of harbouring in his belly an elongated and eroded appendix, with patent opening into the cæcum, full of horribly offensive material, a condition not suspected until exploratory laparotomy had been performed. The removal of this source of continual infection resulted in complete recovery of the patient. Mr. Godlee thinks that in all cases of relapsing appendicitis small abscesses are formed which partially dry up but remain as a possible source of re-infection, and until sterile are a certain cause of continuous poisoning. He spoke of pyorrhœa alveolaris, the disease in which the gums bleed easily, there is a disagreeable taste in the mouth, and often a large quantity of saliva is secreted, containing pus and blood. The breath is foul, and pus containing pockets lie between the gums and teeth. Often the expectoration seems to come from the throat rather than from the mouth. Important in the treatment of this disease, which often occurs in members of the same family, is the removal of the tartar and careful cleaning out of the pus-containing cavities with hydrogen peroxide, chloride of zinc, or essential oils. It may be necessary to remove many teeth. An antiseptic mouth-wash to be used very frequently is a *sine quâ non.* This infective disease may be and often is the cause of fever, rashes, profound septicæmia, purpuric hæmorrhages, bleedings from gums, toxic neuritis, septic gastritis, stomatitis, pharyngitis, tonsillitis, ulcerative endocarditis, pernicious anæmia, dyspepsia, bronchiectasis, and other forms of definite and indefinite ill-health. Possibly, when lardaceous disease occurs without other obvious cause, it may be also due to the buccal suppuration. Cases of empyema in which drainage is free may discharge for years without lardaceous disease; for this reason it is well not to dispense with the drainage-tube early. Pulmonary osteoarthropathy is the result of chronic septic poisoning; leucorrhœa as a cause of septic osteoarthritis in young women is often overlooked. Some patients with chronic tuberculous glands are always ailing although the glands may not be discharging; the patients become robust after the removal of these caseous lumps. Mr. Godlee described a case of osteomyelitis of the femur in which the patient, a boy aged seven years, after passing through a severe attack of acute septic poisoning, during the course of which pus was found and evacuated from the pericardium, lungs, elbow joints, and brain, left the hospital all the wounds healed. But 18 months from the onset of symptoms he died of intracranial suppuration, and a small abscess containing a sequestrium was found at the site of the first incision. Another patient, for 40 years had endured recurrent pyæmic manifestations owing to the fact that two or three small old sequestra had been left embedded in callus after an operation performed for necrosis of the lower end of his femur. Mr. Godlee concluded by impressing on his hearers the importance of getting rid of those little drops of pus, so small and so easily and frequently neglected, which are the cause of such widespread disorders.

[1] Lancet. December 5.

